# Teaching evolution to psychiatrists in Venezuela: comparison with medical students and other medical specialists: a pilot study

**Published:** 2012-09-30

**Authors:** Trino Baptista, Elis Aldana, Félix Angeles, Heidy Delgado

**Affiliations:** 1 Departments of Physiology and Psychiatry, Los Andes University Medical School, Mérida, Venezuela; 2 Department of Biology, Los Andes University Science School, Venezuela; 3 Los Andes University School of Criminology, Mérida, Venezuela; 4 Department of Psychiatry, Los Andes University Medical School, Mérida, Venezuela

## Abstract

**Introduction:**

The teaching of Evolution Theory (ET) in medical programs has received scant attention in the literature. In this report, we first describe the main applications of ET in medicine. Second, we present the evaluation of an interactive seminar on ET given to groups of medical students, psychiatrists, and other medical specialists.

**Methods:**

A two-hour, four-module, interactive seminar was conducted with separate groups of 27 psychiatrists, 15 family doctors, 18 neurologists, 13 physiatrists, 12 internists, and 24 sixth-year medical students without formal training in ET. Their knowledge of ET before and after the seminar was rated on a validated analogical scale (0–12). In addition, the perceived relevance of the information for the participants’ professional activity was assessed.

**Results:**

*S*core averages and medians before the seminar were below 6, suggesting low to moderate knowledge. The students’ scores did not differ significantly from those of the physicians except on the Hominization item, where they scored lower than the physicians (*p* < 0.05). The psychiatrists’ scores did not differ from those of the other groups before the seminar, but after the seminar the increase in their scores on a number of items was significantly smaller than that of the other groups. While all groups scored 10 or more when assessing the relevance of the information, the psychiatrists had the lowest score (*p* < 0.05).

**Discussion:**

The results show the adequacy of short programs to enhance knowledge on ET. This may assist medical educators to develop comprehensive and compulsory courses. Future studies must explore whether psychiatrists are relatively reluctant or ambivalent to accept evolution concepts and proposals.

## Introduction

Even though it is widely acknowledged that Evolution Theory (ET) is a basic science for medicine,^1^,^2^ its teaching is not formally included in any undergraduate medical curricula in Venezuela, and there is only one postgraduate residency program (in psychiatry) that includes it in its training (see below). Evolution concepts may assist psychiatrists in enriching a wide range of professional activities, such as patient and family education,^3^ psychotherapy,^4^–^6^ and research.^7^–^9^

A survey conducted in the United Kingdom in 1997 obtained answers from twenty out of thirty medical schools. Eleven schools included sessions in animal/human evolution in their curriculum and eighteen provided information about population genetics.^10^

Nesse and Schiffman^11^ sent a 30-question questionnaire to each medical school dean in North America and responses were received from 30 schools. Forty eight percent of the deans answered “yes” to the question “At your medical school, is evolutionary biology regarded as important knowledge for physicians?” Only three schools require a course in evolutionary biology as a prerequisite for graduation, and only two have “a distinct course or lecture sequence that presents evolutionary biology as a basic medical science.” We could not find any additional published study on this topic, and this is considered an important drawback in medical education.^11^

Even scarcer is the published information about the teaching of ET in postgraduate medical training around the world. In 2002 we sent a letter to the academic coordinators of all the postgraduate psychiatry training programs in Canada, asking whether their program included a formal course on ET. Twelve out of seventeen coordinators answered our letter, and none provided a formal discussion of ET.^12^,^13^ A symposium on evolutionary medicine for 2^nd^-year medical students was held in May 2010 at the University of Auckland, New Zealand,^1^ and an optional course on evolutionary psychiatry is offered at the University of Barcelona, Spain.

Since 1986, a course about the applications of ET in psychiatry has been provided in the psychiatric residency program in Mérida, Venezuela,^14^ but to the best of our knowledge, no other similar programs have been reported in the literature. The initial program has been revised, modified and published elsewhere.^12^,^13^, ^15^–^17^ This introductory course has not been formally evaluated and such an evaluation is necessary to support the proposal to include a required course on ET in the psychiatric training programs.

In this report, we first describe the main applications of ET in medicine. Second, we present the results of an interactive seminar on ET given to medical students, psychiatrists, and other medical specialists. We compared the level of general knowledge on evolutionary topics of Venezuelan psychiatrists before the seminar with that of the other groups and how this general knowledge changed after the training session.

We hope we will thus open a forum and improve the teaching of ET in psychiatry and other branches of medicine.

### Applications of Evolution Theory in medicine

Nesse and Schiffman^11^ identified sixteen key topics in evolutionary biology that may be considered fundamental subjects for teaching ET in medicine. In a formal educational program the relevance of these key topics for the specific medical specialty should be emphasized (Table 1).

The course on the Application of Evolution in Psychiatry in Venezuela^14^ is compulsory and has been taught in the first semester of the residency since 1986. It includes a weekly 3-hour session during 24 weeks. In addition to the topics covered in the seminar (Appendix 1), two sessions are devoted to the key concepts of Evolutionary Psychology.^1^,^18^–^20^ The final evaluation is the oral presentation of a comprehensive analysis of selected mental disorders according to the models of Tinbergen’s four questions,^1^ Stevens and Price,^21^ and Brüne.^22^

In a recent synthesis, Nesse^8^ proposed ten questions to assist researchers in evolutionary studies of disease vulnerability. This model, which requires a more advanced level of knowledge in evolution topics, is used when discussing specific research protocols in the Psychiatric Department of Los Andes University.

## Method

### Assessment of the level of knowledge on evolution topics

In 2011 we introduced a 2-hour interactive seminar on ET under the sponsorship of the Department of Physiology at Los Andes University Medical School (Mérida, Venezuela). Separate identical sessions were conducted with 27 psychiatrists (from Maracaibo, Zulia state, Venezuela), 15 family doctors, 18 neurologists, 13 physiatrists, 12 internists, and 24 sixth-year medical students attending a clerkship. The seminar used the development and pathology of the human spinal column as a key example particularly suitable for evolutionary analysis. The participants were selected because we considered that this type of physicians and students assist patients with chronic diseases that are appropriate for evolutionary analysis. With the exception of psychiatrists, all the attendees worked or studied at Los Andes University. No group had ever received any formal training on evolution topics.

### Content of the seminar

The seminar consisted of four 30-minute modules:

- Module 1: Life, History of the Evolution Theory, The Human Lineage^1^,^23^,^24^
- Module 2: Mechanisms, Outcomes and Challenges to Evolution Theory^1^,^25^–^30^
- Module 3: The Process of Hominization: Focus on the Vertebral Column^1^,^31^–^35^
- Module 4: Applications to Clinical Medicine and Research^1^,^2^,^3^,^8^,^22^,^36^–^38^

A detailed list of the topics covered in each of the four modules can be found in Appendix 1.

#### 1. Objectives

- To assess the impact of a two-hour seminar on the level of knowledge about key evolution topics on psychiatrists, other medical specialists, and medical students.
- To compare the level of general knowledge on evolution topics among psychiatrists and the other groups before and after the seminar.
- To describe the participants’ opinion about the relevance of the seminar for their professional education.

#### 2. Procedure

- Before and after the seminar the participants completed an anonymous questionnaire with information about their age and gender, and answers to the eleven questions below (Table 2). Every topic was qualified with a visual analogical scale, where 0 corresponded to “no information at all” and 12 to “much information”.
  The 11-item questionnaire was evaluated by four experts (two biologists and two psychiatrists) to assess its content validity (CV), yielding a CV coefficient of 0.89 (error = 0.003) which can be considered “high”. Reliability was assessed in an independent sample of ten psychiatric residents. The Cronbach’s Alpha coefficient for internal consistence was 0.95 (*df* = 10, 98), *p* < 0.01.
- After the seminar, using the same analogical scale, the participants reported how their knowledge about the specific topics had changed. They also answered the following question using a similar scale: *“How relevant do you think the information is for your professional activity?”*
- The score averages were compared among the groups with two-tailed *t-*test for unrelated samples and two-way ANOVA (when normally distributed). The Kruskal-Wallis and median tests were used for non-normally distributed data.

In the post-seminar assessment, the pre-seminar scores for each specific item were the covariates in the General Lineal Model analysis. The influence of age was analyzed with a bivariate correlation analysis (age vs. scores for each item). A two-tailed *t-*test was used to assess the role of gender. Results were considered significant when *p* < 0.05.

## Results

Table 3 describes the basic demographic features of the participants. Sixty-four percent were women; the psychiatrists were significantly older than the medical students, the physiatrists and the internists.

Two types of analysis were conducted before and after the seminar: the first analysis compared the scores obtained on the eleven questions (between 0–12) between all the physicians and the medical students (two-group analysis, Table 4). The second analysis compared the scores among the psychiatrists and the other groups (six-group analysis, Tables 5 and 6).

### Evaluation before the seminar

#### Two-group analysis

Most score averages and medians were below 6. Physicians obtained higher scores on questions 1–4, 9 and 10, whereas students obtained higher scores on questions 5–8. Both groups obtained similar scores on question 11. Only for question 9 (the concept of Hominization) the comparisons were statistically significant (Table 4).

#### Six-group analysis

**Figure 1:**
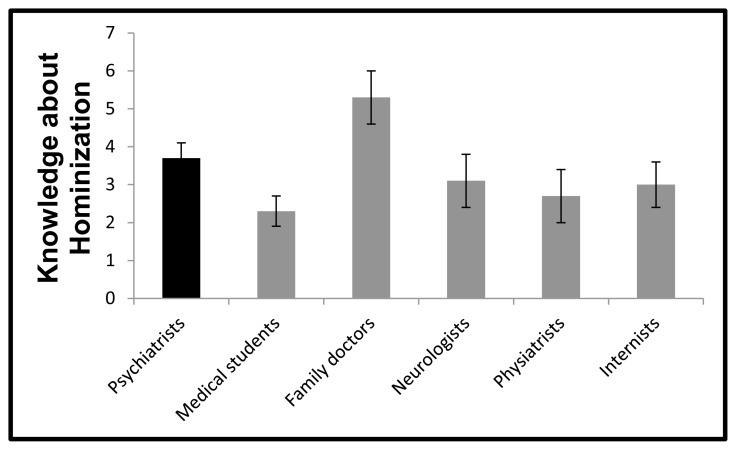
Pre-seminar scores on the level of knowledge about the process of Hominization Values represent mean ± standard error. Global analysis: *f* (5, 108) = 3.4, *p <*= 0.001; family doctors vs. medical students: *p* < 0.01.

Question 9 (Hominization) was again the only item where *between-group* differences were significant. The family physicians displayed the highest score (*p* < 0.01 in the overall analysis). *The post-hoc* analyses were significant only for the medical students (*p* < 0.01) (Figure 1). The rest of the data are not shown.

### Evaluation after the seminar

#### Two-group analysis

When asked, “*How did your knowledge change about specific topics?”,* a significant increase in the scores on all topics was observed in the whole group of participants (*p* < 0.001, data not shown). The increase ranged from 4.6 to 7.2 points and did not differ between the medical student group and the physician group on any of the items (*p* > 0.05).

#### Six-group analysis

Changes on the items “Common Ancestor” (n∘ 2), “the History of the Human Lineage” (n∘ 3) and “Evolution as a Feature” (n∘ 4) showed significant differences among the six groups. On all of these items, the psychiatrist group showed the smallest level of change (Table 5).

Additionally, we compared the change in the total scores of all items, with the pre-seminar values as covariates. Again, the psychiatrists reported the smallest global change (mean ± *SD*), which did not reach statistical significance: psychiatrists: 5.1 (*SD* = 0,4); medical students: 6.2 (*SD* = 0.4); family doctors: 5.7 (*SD* = 0,5); neurologists 6.4 (*SD* = 0.5); physiatrists: 6.9 (*SD* = 0.6); internists: 6.2 (*SD* = 0.6): *f* (5, 89) = 1.5, *p* = 0.19.

### Relevance for professional activity

When asked *“How relevant was the information for your professional activity?”,* all participants scored a median of 11 and an mean of 10.7 (*SD* = 1.6). Taking into consideration that the scale’s top was 12, these results point to a positive evaluation.

**Figure 2:**
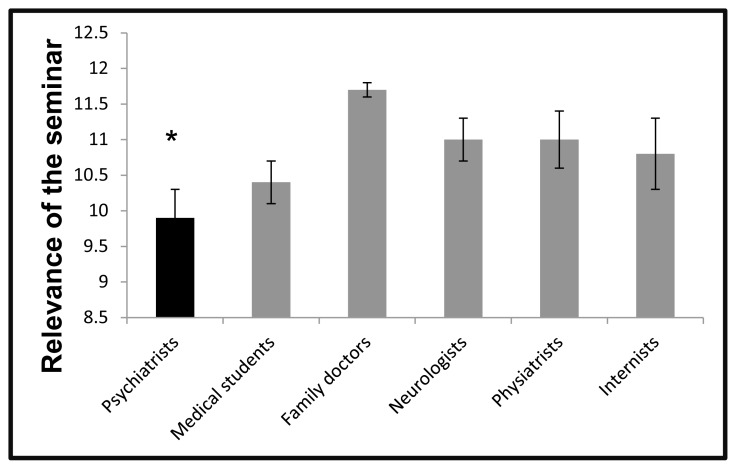
Opinion of the Six Groups about the Relevance of the Information for their Professional Activity Kruskal-Wallis *χ*^2^ (5) = 12.9, *p* < 0.05; (*) Wilcoxon test, psychiatrists vs. family doctors, *p* < 0.01.

Surprisingly, the psychiatrists had a median of 10 and a mean of 9.9 (*SD* = 1.9), which was the lowest score of all the groups (*p* < 0.05, Figure 2).

### Influence of age and gender

#### Before the seminar: 6-group-analysis

A significant (positive) correlation between age and the scores on items 5 and 7–11 (*p* < 0.05) was found in the psychiatrist group only.

The scores were significantly higher for males in the neurologist group on item 8 and the internist group on items 5, 8 and 11 (*p* < 0.05).

#### After the seminar: 6-group analysis

Age and the change in knowledge level on specific items or the perceived relevance of the seminar were not significantly correlated in any group (*p* > 0.05).

The change in knowledge level was significantly higher in female family doctors (item 2), neurologists (item 7) and internists (items 5,6,8,11).

## Discussion

Evolution is a basic science in medicine, and it should be formally incorporated in the medical curricula.

In Venezuela, no undergraduate medical program and only one postgraduate course (in psychiatry) includes a course on ET as a prerequisite for matriculation.^14^ This is probably the case in most countries, but scarce information is published on this topic. In Venezuela, a brief introduction to ET is provided in High School. At Los Andes University Medical School, one of the top academic centers in the country, ET is not included in the General Biology courses. However an evolutionary analysis of social organization is imparted in an optative subject named The History of Western Paradigms.

The authors will request the inclusion of several modules of formal information about ET in the undergraduate medical curriculum in Venezuela. In addition, the existing course for psychiatry residents^12^,^13^,^14^,^15^,^16^ will be offered to all twelve residency training programs in psychiatry in Venezuela. This might stimulate the residency programs in other specialties to also develop specific programs on ET.

Even though the questionnaire for evaluation showed a good content validity and reliability, the assessment of the seminar outcomes has several limitations: 1) the physician and student samples were not probabilistic; 2) the samples did not encompass all the medical schools in the country; 3) ideally, there should be a follow-up study of the same subjects in the undergraduate and postgraduate medical courses; 4) the information was deliberately condensed and the evaluation was rather crude, by having assessed the participants’ *opinion* about their knowledge on specific items instead of their actual knowledge. Therefore, the weaknesses and strengths detected in the evaluations cannot be generalized and should be considered strictly preliminary. However, the authors consider that in view of the current state of medical education on ET, a simple procedure was desirable.

The pre-seminar evaluation showed that the general level of knowledge on ET items was low to moderate. In six out of eleven items, the physicians had higher scores than the students, and in four it was the opposite. No single pattern is apparent that any of the questions favored either one of the two groups. Interestingly, in the psychiatrist group only knowledge on specific items increased with age (see below for further discussion). In general, the pre-seminar scores did not differ between both genders.

When discriminating by medical specialty in the pre-seminar evaluation, the psychiatrists’ scores were not significantly different than the scores of any other group, but the family doctors had the highest score on the hominization item. This result suggests that these specialists might be particularly suitable and open to education in evolution. Given the widespread influence of family physicians on the general health of the population, they may be effective promoters of evolutionary ideas among patients, family, and other physicians.

The significant increase in knowledge about the evolution topics observed in the whole sample, and independently of age, confirms the adequacy of short and specifically designed programs to improve medical education. The knowledge level of female family doctors, neurologists and internists increased more than that of males on specific items.

The psychiatrists’ post-seminar scores on three topics of the first module (n∘ 1, Common Ancestor; n∘ 3, the Human Lineage and n∘ 4 Evolution as a Feature of Living Beings) showed a significantly smaller increase than those of the other groups. Furthermore, psychiatrists had the lowest score on the relevance of the information for their professional activity. Still, their scores were clearly positive. Interestingly, only in the psychiatrist group was there a positive and significant correlation between age and the level of knowledge on 6 items before the seminar. These apparently contradictory results should be explored in future studies to assess whether they imply a relative reluctance or ambivalence towards evolutionary ideas among psychiatrists.

## Figures and Tables

**Table 1:** Key topics for teaching ET in medicine

Antibiotic resistance Virulence evolution Population genetics Selection for disease genes Mutation selection balance Levels of selection Host–pathogen arms race Novel environment causing disease Trade-offs Comparative anatomy Defense regulation Life history evolution Design flaws from path dependence Primate phylogeny Kin selection Proximate/ultimate distinction

**Table 2:** Pre and post-seminar questions

**Pre-seminar**: During your professional education, how much information did you receive about…? **Post-seminar**: After this seminar, what is your level of knowledge about...?
The scientific concept of life and the history of Evolution Theory. The notion that all life is connected (Common Ancestors). The history of the human lineage. Evolution as a feature of living beings. Biological and social components of human evolution. The concept of biological adaptation. Adaptive value of symptoms and disease. Design constraints and trade-offs in human disease; vulnerability to disease. The process of Hominization. Proximate and ultimate causes of disease: the vertebral column as an example. Evolutionary basis for hygiene and prevention.

**Table 3:** Distribution of gender and age of participants

Group	Gender *n* (%)		Age (years)
	Women	Men	Mean (*SD)*
**Psychiatrists (n = 27)**	18 (66.6)	9 (33.3)	52 (9)
**Family doctors (n = 15)**	12 (80)	3 (20)	50 (4)
**Neurologists (n = 18)**	11 (61)	7 (39)	53 (6)
**Physiatrists (n = 13)**	10 (76.9)	3 (23.1)	37 (11)*
**Internists (n = 12)**	6 (50)	6 (50)	37 (11)*
**Medical students (n = 24)**	13 (54.1)	11 (45.9)	23 (1)*

*F* (5, 82) = 37.5, *p* < 0.001, significantly younger than the psychiatrists

**Table 4:** Scores of the pre-seminar evaluation in medical students and of all the physicians

Questions	Group	Scores	
		Mean (*SD*)	Median
1. The origin of life	Physicians Students	3.9 (2.3) (a) 2.9 (2.2)	3 2

2. Common ancestors	Physicians Students	4.4 (2.5) 3.9 (2.8)	4 3.5

3. Human lineage	Physicians Students	3.8 (2.4) 3.1 (2.9)	3 2

4. Evolution as a feature	Physicians Students	4.7 (2.8) 4.6 (2.1)	5 4.5

5. Biological and social evolution	Physicians Students	5.0 (3.1) 5.2 (2.7)	5 5

6. The concept of adaptation	Physicians Students	4.9 (2.9) 6.0 (2.8)	5 6

7. Adaptive values of symptoms	Physicians Students	4.9 (2.7) 5.5 (3.1)	5 6

8. Design compromises	Physicians Students	5.8 (3.0) 6.4 (3.3)	5 6

9. Hominization	Physicians Students	3.6 (2.5) (b) 2.3 (2.2)	3 (c) 1

10. Proximate and ultimate distinction	Physicians Students	3.9 (2.9) 3.1 (2.2)	3 3

11. Hygiene and prevention	Physicians Students	5.4 (3.2) 5.4 (3.0)	5 5

*t* (107) = 1.8, *p*= 0.06;

(b) *t* = 2.4, *p* < 0.05;

(c) Median *χ*^2^ (1) 0 2.5, *p* = 0.06.

**Table 5:** Change in knowledge on specific items after the seminar

Group	Items		
	Common ancestor *Mean* (*SD*)	Human lineage *Mean* (*SD*)	Evolution as a feature *Mean* (*SD*)
**Psychiatrists**	5.2 (0.3) (a)	5.7 (0.3) (b)	4.9 (0.3) (c)

**Family doctors**	6.3 (0.4)	7.3 (0.4)	6.6 (0.4)

**Neurologists**	5.9 (0.4)	6.7 (0.4)	5.7 (0.4)

**Physiatrists**	6.3 (0.5)	6.9 (0.5)	6.2 (0.4)

**Internists**	6.8 (0.5)	7.0 (0.5)	5.9 (0.4)

**Medical Students**	6.4 (0.3)	6.5 (0.3)	5.7 (0.3)

Values represent adjusted means after covariate analysis.

(a) *F* (5, 107) = 2.7, *p* < 0.05; *p* < 0.05 vs. internists;

(b) *F* = 2.6, *p* < 0.05; *p* < 0.05 vs. family doctors;

(c) *F* = 3.1, *p* < 0.01; *p* < 0.01 vs. family doctors.

## Appendix 1 Detailed content of the Modules

- **Module 1: Life, History of Evolution Theory, The Human Lineage**^1^,^23^,^24^
  a.1) The scientific definition of life.
  a.2) The history of Evolution Theory: Jean Baptiste Lamark, Erasmus Darwin, Charles Darwin, Alfred Wallace.
  a.3) The contribution of neo-Darwinism: geology, ethology, sociobiology, molecular biology, current challenges (see module 2).
  a.4) Common ancestors (LUCA as an example).
  a.5) The history of the human lineage: Linnaeus, Haeckel, Chaton, Copeland, Whitakker, Woese.
  a.6) The genus Australopithecus and Homo in geological time.
- **Module 2: Mechanisms, Outcomes and Challenges to Evolution Theory**^1^,^25^–^30^
  b.1) Mendelian segregation.
  b.2) The mechanisms of evolution: natural selection, genetic drift (the case of porphyria variegata and huntington chorea in South Africa), sexual selection, cultural evolution.
  b.3) The concept and calculation of fitness (recent insights into the features that increase or decrease fitness in humans).
  b.4) The concept of genetic load.
  b.5) The consequences of evolution: adaptation and exaptation (the case of haemoglobin), molecular co-evolution (the case of myxomatosis in rabbits, vertical transmission in Chagas’ disease; dengue as an example of absence of molecular co-evolution).
  b.6) Current challenges to Evolution Theory: horizontal transference of genes (the case of the trypanosoma cruzi), molecular drive (the case of the expression of the liver genes in human embryos and in adults), units of evolution (individual, group, kin selection), the hox genes, neutral molecular evolution.
  b.7) The human genome in geological time.
- **Module 3: The Process of Hominization: Focus on the Vertebral Column**^1^,^31^–^35^
  c.1) The concept of Hominization.
  c.2) The vertebral column as an example: its evolution along the phylogenetic tree. the transition to bipedalism.
  c.3) The concurrent changes in other bodily systems: focus on the brain, skull, pelvis, hands and feet.
  c.4) Adaptive consequences of the spinal column evolution: focus on thermoregulation and the development of handiness.
  c.5) The concept of trade-off. How the evolution of the column made us vulnerable to disease.
  c.6) How the above-mentioned knowledge may assist physicians with hygiene and prevention of spinal column diseases.
- **Module 4: Applications to Clinical Medicine and Research**^1^,^2^,^3^,^8^,^22^,^36^–^38^
  d.1) The four questions of Tinbergen: proximate causes, ontogeny, function (adaptive value) and ultimate causes (phylogeny).
  d.2) Features that often coexist in a specific disease: direct effects of the pathogen; useful defenses; inadequate consequences of defenses; impact of genes; historical legacies; design constraints; trade-offs; the influence of current and past environments.
  d.3) Sickle cell anemia as a key example.
  d.4) Bipolar disorder: how can Evolution Theory assist us in the search for pathogenesis and novel treatments.
    d.4.1) **Proximate causes**: neurotransmitters, neuroregulators, hormones, brain circuits.
    d.4.2) **Ontogeny of bipolar disorders:** childhood onset; the search for hygiene and prevention: genetic counseling, sleep hygiene, management of stressors and relapses.
    d.4.3) **What is the function of the phenomena?** The proposed adaptive value of depressive and manic symptoms. How did it go wrong? Hypothesis for the high prevalence of type 2 diabetes mellitus, metabolic syndrome, migraine, and inflammation. Relevance in the search for novel treatments.
    d.4.4) **Ultimate causes (phylogeny):** complex interaction among biological rhythms, mood and energy balance regulation, social interaction, genes, etc. Animal models: focus on kindling and thyrotrophin- releasing hormone (TRH).
